# Health disparities in Russia at the regional and global scales

**DOI:** 10.1186/s12939-021-01502-6

**Published:** 2021-07-13

**Authors:** Natalia Shartova, Vladimir Tikunov, Olga Chereshnya

**Affiliations:** Faculty of Geography, Lomonosov Moscow State University, Moscow, 119991 Russia

**Keywords:** Health indicators, Typological ranking, Multivariate analysis, Spatial heterogeneity

## Abstract

**Background:**

The capacity for health comparisons, including the accurate comparison of indicators, is necessary for a comprehensive evaluation of well-being in places where people live. An important issue is the assessment of within-country heterogeneity for geographically extensive countries. The aim of this study was to assess the spatial and temporal changes in health status in Russia and to compare these regional changes with global trends.

**Methods:**

The index, which considers the infant mortality rate and the male and female life expectancy at birth, was used for this purpose. Homogeneous territorial groups were identified using principal component analysis and multivariate ranking procedures. Trend analysis of individual indicators included in the index was also performed to assess the changes over the past 20 years (1990–2017).

**Results:**

The study indicated a trend towards convergence in health indicators worldwide, which is largely due to changes in infant mortality. It also revealed that the trend of increasing life expectancy in many regions of Russia is not statistically significant. Significant interregional heterogeneity of health status in Russia was identified according to the application of typological ranking. The regions were characterized by similar index values until the mid-1990s.

**Conclusions:**

The strong spatial inequality in health of population was found in Russia. While many regions of Russia were comparable to the countries in the high-income group in terms of GDP, the progress in health was less pronounced. Perhaps this can be explained by intraregional inequality, expressed by significant fluctuations in income levels.

**Trial registration:**

Not applicable.

## Background

The capacity for population health comparisons is critical for a comprehensive assessment of well-being across different areas. Various metrics can be used to assess the health status of a region during different periods of socioeconomic development, including indicators of morbidity, temporary and permanent disability, number of hospitalizations, and standardized mortality. The issue of creating integral indicators for a comprehensive assessment of population health has not lost its relevance. Since the 1970s, one of the most common indicators has been quality-adjusted life years (QALYs), which combine life expectancy and quality of life [1]. In the early 1990s, the QALY model served as the basis for the development of another health indicator – disability-adjusted life years (DALYs). The DALY model measures the disease burden through the introduction of disability rates [2]. Currently, these indicators are used in most economic assessments, especially when health cost-benefit analysis is an integral part of decision-making [3]. While the age profiles of disability for DALY calculations are generally straightforward, the actual calculations can be relatively complex [4]. In addition, statistics on disability are not always available for all countries and regions, which significantly complicates the use of these indicators in interregional analysis.

А large number of health indices have been developed. Their use depends on the objectives and scope of the research, as well as the target audience. More detailed information on the existing indices can be found in reviews [5–7]. Traditionally, life expectancy and infant mortality have been used as some of the most objective health indicators [8–10]. They are important indicators of a nation’s health and well-being and can reflect human development and social progress [11].

According to the World Health Organization, there are positive tendencies of life expectancy increasing and infant mortality reducing in the world. Global life expectancy has increased by more than 6 years over the last 20 years [12]. This trend has been gradual but steady, although life expectancy is highly influenced by a variety of factors. For example, one of the most significant and unexpected events was the stagnation and decline in life expectancy in Russia and other former communist countries in the second half of the twentieth century [13]. Recent catastrophic incidents such as the Rwandan genocide and North Korean famines or escalating mortality due to different infectious diseases, including HIV or COVID-19, can also affect and stagnate the longevity worldwide [14].

Difference in longevity between countries and regions has remained significant. In 2019 life expectancy at birth varied from 53.1 years in Central African Republic to 84.26 years in Japan [12]. Large spatial differences in life expectancy at birth were found in Latin American cities, including the capitals – Panama City, Santiago, Mexico City and Buenos Aires [15]. In-country inequalities can be indicators of a nation’s health, as well as social justice and societal equity [16]. Comparing the length of life in the regions of Sweden and Finland has shown that regional inequalities can exist in high-income countries with high life expectancy and can be extremely persistent [17]. A north-south gradient of life expectancy has been found in England and Wales that was mainly attributable to variations in deprivation status [18]. Difference in life expectancy between mainland population and residents of islands was identified in Croatia. Life expectancy on islands was higher and comparable with the length of life in neighboring Slovenia [19].

Inequalities in life expectancy by sex generally have also increased over time due to uneven progress in health in association with social factors [14]. This problem has been relevant for Russia for many years. In 1994, when the minimal rate of life expectancy since 1960 was identified, life expectancy at birth was 57.4 years for men and 71.1 years for women [20]. According to the study by Pietila and Rytkonen [21] the gender difference in mortality and life expectancy in post-Soviet Russia could be linked not only to behavioural factors, including hazardous alcohol consumption, but also to structural conditions and changes in Russian society. Gradual adaptation to a new socio-economic environment has resulted in life expectancy increasing, however, the difference between the sexes has still remained. In 2019 the difference by sex was about 10 years (68.18 years for men and 78 years for women) [12]. However, an increase in life expectancy does not mean an increase in healthy life expectancy. This indicator has lagged behind and depends on public health [11].

One of the main features of the post-Soviet Russian health crisis was the high male mortality rate, especially in the working-age group due to cardiovascular diseases and external causes (accidents, injuries, etc.) [22]. Since the early 2000s, mortality has progressively reduced in Russia primarily due declining deaths from these main causes [23, 24]. However, injuries and injury deaths rates, cardiovascular mortality and morbidity have remained among the highest in Europe. In addition to traditional determinants of health (age, income, marital status, settlement type, etc.), chronic diseases are important in the public health context of Russia. More than 40% of citizens currently live with multi-morbidity and 86% of all deaths are due to chronic diseases [25]. According to the data from Russian Longitudinal Monitoring Survey provided by the Higher School of Economics [26], the most common chronic illness reported by men and women in 2014 was high blood pressure, while cancer was identified as the rarest disease [25].

In contrast to this information, the ambitious “National goals and strategic objectives for development of the Russian Federation for the period up to 2024” was announced in 2018 [27]. One of the priorities was declared as achieving life expectancy of 78 years by 2024, and 80 years by 2030, exceeding the current value by 5 and 7 years, respectively. This requires not only a quite distinctive health-sector initiative, but a systematic monitoring of patterns of population health and learning from the best international practice.

There might be several potential problems in achieving this goal. One important issue concerns the assessment of in-country heterogeneity in geographically expansive countries. Considering that patterns of regional health disparities have been clearly presented in the group of former communist countries of Central and Eastern Europe and divergence became more heterogeneous over time [28, 29], this might be quite relevant for Russia. The problems of generalizing, comparing, and interpreting of statistical data are especially noticeable when analysing territories on various scales. Moreover, index complexity does not guarantee obtaining comparable results for different territories. In any case, there is no official index in Russia to date, and none of the existing indices are used in the decision-making system [30].

The main problem concerns the availability and quality of statistical data. For example, mortality statistics in Russia were classified for a long time, and the data appeared for public use in 1988. A long-term series of statistics on causes of death became available eight years later, when demographers reconstructed the data for 1965–1994 [31]. At present, statistics of multiple causes of death, depersonalized information on the socio-economic status of the deceased individuals, as well as their citizenship and ethnicity are still unavailable for research purposes [32]. Health statistics are also published in an extremely aggregate form and are available at the regional level only. A great deal of information remains in original forms at the places of registration. Municipal statistics are generally very poorly developed and represent the situation only in two main cities, Moscow and St. Petersburg [33]. Understanding the causes of health inequalities requires considering the social determinants of health [34], however, many indicators about population lifestyle and social status are simply not available in Russia.

Another serious problem is related to coding practice according to International Classification of Diseases (ICD-10) and associated errors in diagnoses and particularly cause-of-death coding. In Russia, individual medical practitioners manually code the causes of death for statistical purposes [35]. They do not experience a real need for mortality statistics at population level and do not always take responsibility for the accuracy of coding [36]. There are many questions about the high proportion of undetermined causes of death including “event of undetermined intent”, “ill-defined and unknown causes of mortality” “cardiomyopathy, unspecified”, etc. This is possible concerned so-called “socially significant causes of death” (murders, suicides, alcohol, and drug poisoning) and causes targeted to reductions, primarily cardiovascular diseases [32]. A recent example of a problem with coding in Russia appeared during the Covid-19 pandemic, when the practice of attributing some deaths to other causes in people who test positive for the virus, against World Health Organization advice was applied [37].

Global, regional, national, and subnational data for socioeconomic development and population health indicators including morbidity, temporary disability, disability, number of hospitalizations and standardized mortality are not always accurate and available across the world [38, 39]. This impedes the creation of complex indices for population health assessment, as well as comparison with regard to the international aspect. This study is aimed at assessing the spatial and temporal changes in the health of the population in Russia and comparing the regional changes with global trends. We deliberately chose the simplest and most unified indicators to ensure the possibility of reliable inter-comparison and eliminate the many problems linked to incomparability.

## Methods

### Study area

The Russian Federation is a country in the northern part of Eurasia, covering an area of over 17 million km^2^ and having a population of over 146 million people. Russia is territorially divided into 85 federal units - oblasts, republics, krais, autonomous oblasts, autonomous okrugs (hereinafter regions), and federal cities. The population density varies by region from 0.07 to 171.5 people/km^2^, with the exceptions of the federal cities of Moscow, St. Petersburg, and Sevastopol,^1^ whose population densities are 4852, 3846, and 492 people/km^2^, respectively.

### Data sources

We used an integral index to measure the health of populations which integrates objective indicators of population health: infant mortality rate and life expectancy at birth for men and women. We called it Public Health Index (PHI) due to life expectancy and infant mortality are basic statistics that indicate public health achievements and social development, including the health system, infrastructure, and vital statistics [40].

The use of these simple indicators offers several important advantages: relevant data are available for almost all countries, expert assessment is not required, and the indicators are reliable [41].

The data sources for the infant mortality rate and life expectancy were the World Bank database [42] for countries worldwide and the Federal State Statistics Service “Rosstat” [43] for the regions (85 federal units) of Russia. The data covered the period from 1990 to 2017. Additionally, data on GDP values were used as the driver of health to compare the health status to the income level. The information was taken from the World Bank database [44] and “Rosstat” [43].

Several problems of data quality in Russian statistics are given in the introduction. This should be considered while interpreting the results. Nevertheless, a unified methodology of Rosstat for collecting and aggregating data allows them to be used for cross-country analysis and international comparing. World Bank relay on professional standards in the collection and compilation of data to ensure the data consistency and accuracy. Developing countries receive support to improve the capacity and efficiency of national statistical systems. Thus, this data is widely used in population health assessment and human development studies [45–48].

### Index calculation and typological ranking

For calculation of the PHI, an evaluative algorithm was developed by one of the authors [49]. The normalization of initial indicators is achieved by the following formula:

$$ {\hat{x}}_{ij}=\frac{\left|{x}_{ij}-{\overset{\circ }{x}}_j\right|}{\mid {}_{\mathit{\max}/\mathit{\min}}{x}_j-{\overset{\circ }{x}}_j\mid },i=1,2,3,\dots, n;j=1,2,3,\dots, m; $$

where $\overset{\circ }{x}$ is the worst value (for each indicator), in terms of the impact on the population health in the regions of Russia (the maximum infant mortality rate, lowest life expectancy);

_max/min_*x* is the most different from the $\overset{\circ }{x}$ values of n countries and regions.

m is the number of indicators used for the calculations (3).

The ranking is carried out by comparing all territorial units on a conditional basis, characterized by values of $\overset{\circ }{x}$. If there are reasonable weights for the indicators, they can also be included in the formula of normalization, but in our calculations, the weights were the same due to the contribution of infant mortality and life expectancy in health loss can vary over time and countries may also be at different phase of the epidemiological transition.

When the normalized values ${\hat{x}}_{ij}$ are reduced to comparable forms, they can be simply summarized to obtain the PHI.

$$ {\hat{\mathrm{S}}}_{\mathrm{i}}=\sum \limits_{j=1}^m\frac{\left|{\mathrm{x}}_{\mathrm{i}\mathrm{j}}-{\overset{\circ }{x}}_j\right|}{\mid {}_{\max /\min }{\mathrm{x}}_{\mathrm{j}}-{\overset{\circ }{x}}_j\mid },\mathrm{i}=1,2,3,\dots, \mathrm{n};\mathrm{j}=1,2,3,\dots, \mathrm{m}; $$

Received values $\hat{\ \mathrm{S}\ }$ characterize the estimated position of the countries and regions of Russia. The algorithm can be very parsimonious. The ranking is carried out by comparing all territorial units on a conditional basis, characterized by values of $\overset{\circ }{x}$. This is done using the Euclidean distance as a measure of the proximity of all territorial units to a conditional basis (a worst-case value $\overset{\circ }{x}$ throughout a range of indicators). We believe that improvement experiences should start with the worst regions / countries, thus, we focused on the worst regions in order to highlight “hotspots” for further improvement of the situation.

The ranking was performed by comparing all indicator values attributed to territorial units with conditional $\overset{o}{x}$ values. Euclidean distances (*d°*) were used for the ranking procedure. (*d°*) is the measure of the closeness of all territorial unit values to the worst values of conditional ($\overset{o}{x}$) regarding the whole set of indicators.

$$ {d}_{ik}=\sqrt{\sum \limits_{j=1}^m{\left({\hat{x}}_{ij}-{\hat{x}}_{kj}\right)}^2},i=1,2,3,\dots, n;j=1,2,3,\dots, m; $$

The algorithm required the preliminary processing of the data array using the method of principal component analysis to orthogonalize and convolute the system of indicators. The received data of the column vector *d°*presenting assessment characteristics were additionally normalized for convenience using the following formula:

$$ \hat{d}{}_i{}^o=\frac{d_i^o-{{}_{\min }d}^o}{{{}_{\max }d}^o-{{}_{\min }d}^o}, $$

$$ \mathrm{i}=1,2,3,\dots, \mathrm{n}. $$

*d°*varies from zero to one. Zero corresponds to the worst integrated assessment, and one corresponds to the best.

The algorithm also enabled the detection of homogeneous territorial groups in the assessment. This was done via a partition of the corresponding ranked values of Euclidean distances into homogenous groups. The allocation procedure for these groups was multivariant and enabled them to receive a spectrum number of homogenous groups of territorial allocation variants. Allocation quality was assessed using canonical correlation coefficients as well as absolute (*A*_*k*_) and relative (*O*_*k*_) coefficients of heterogeneity:

$$ {A}_k=\frac{100\left\{\sum \limits_{k=1}^K\sum \limits_{j=1}^n\sum \limits_{i=1}^n{\left[\sum \limits_{p=1}^P{\left({x}_{ip}-{x}_{jp}\right)}^2\right]}^{1/2}{I}_{ik}{I}_{jk}\right\}}{\sum \limits_{i=1}^{t_{max}}{\left[\sum \limits_{p=1}^P{\left({x}_{ip}-{x}_{jp}\right)}^2\right]}^{1/2}}, $$

$$ \mathrm{k}={t}_{min},{t}_{min}+1,\dots, {t}_{max}; $$

$$ {O}_k=\frac{100\left\{\sum \limits_{k=1}^K\sum \limits_{j=1}^n\sum \limits_{i=1}^n{\left[\sum \limits_{p=1}^P{\left({x}_{ip}-{x}_{jp}\right)}^2\right]}^{1/2}{I}_{ik}{I}_{jk}\right\}}{\sum \limits_{i=1}^{t_{max}}\sum \limits_{j=1}^n\sum \limits_{i=1}^n{\left[\sum \limits_{p=1}^P{\left({x}_{ip}-{x}_{jp}\right)}^2\right]}^{1/2}{I}_{ik}{I}_{jk}}, $$

$$ \mathrm{k}={t}_{min},{t}_{min}+1,\dots, {t}_{max}-1 $$

where k is the number of identified groups, p is the number of orthogonalized coefficients to calculate distances, *t*_*max*_, *t*_*min*_ are the maximal and minimal number of groups, and *I*_*jk*_ is a binary indicator pointing to the presence (1) or absence (0) of the territorial unit in group *k*.

A sharp increase in the absolute or relative coefficients of heterogeneity with a decrease in the number of identifiable clusters indicates an increase in heterogeneity within the identified clusters, while a smooth increase in the coefficients is a sign of a uniform increase. The threshold followed by a sharp increase in heterogeneity can be optimally taken as the final number of clusters. The algorithm was originally described in [49] and has been tested in previous studies [30, 40, 50]. In this study, the dynamics of health status in Russia and in the world for 1990–2017 were revealed according to the calculation of the PHI.

### Trend analysis

The observed changes in health status index components were analysed. The Mann-Kendall nonparametric statistical test was used to assess the significance level of trends, and the Sen slope coefficient was used to assess the rate of change [51]. These statistical methods can be applied even if the time series does not follow a normal distribution [51, 52]. The statistical significance of changes was obtained for each territorial unit (countries at the global level and regions of the Russian Federation) and each parameter. Results with a *p*-value < 0.05 were considered statistically significant. The specific value of Sen slope coefficient depends on the values of estimated variables. A positive value indicated an upward trend and a negative value indicated a downward trend in the time series for each territorial unit.

Index calculation and typological ranking was made by algorithm, elaborated by one of the authors [49]. Trend analysis was performed by R software (package ‘trend’). All results were displayed using QGIS software. The gradations for each parameter are based on natural intervals (Jenks natural breaks) with further manual adjustments [53].

## Results

### Health status dynamics

**Fig. 1:**
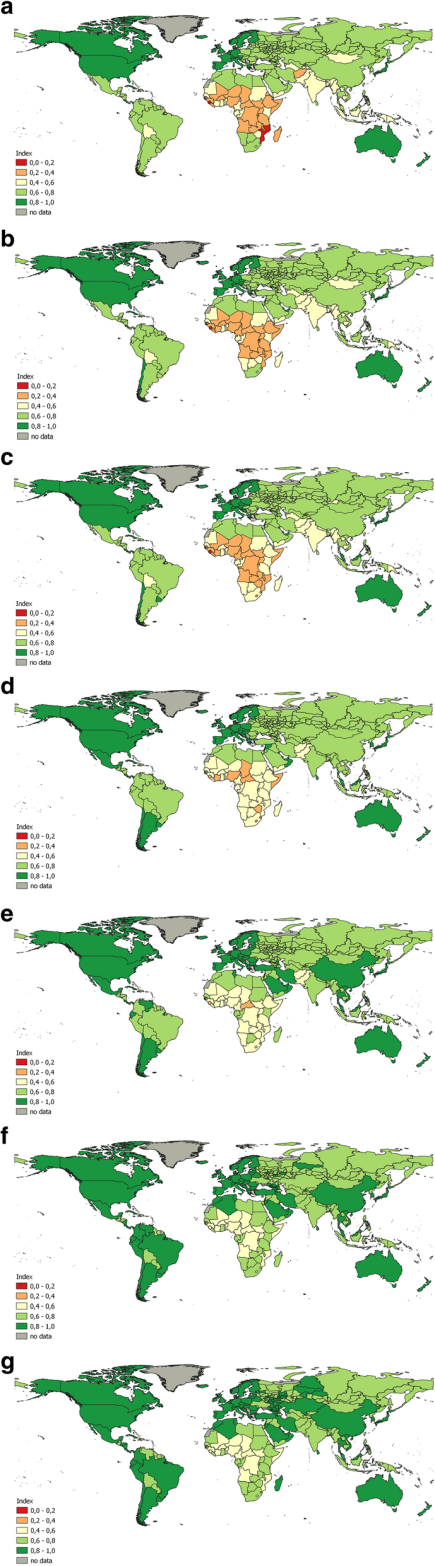
Changes in population health identified by PHI in the regions of Russia and the world during 1990–2017; a) 1990; b) 1995; c) 2000; d) 2005; e) 2010; f) 2015; g) 2017

Globally, there is a trend of an increase in PHI values over the past 20 years (Fig. 1). During this time, the lowest rate was in Sierra Leone (0.14 in 1990 and 0.44 in 2017). In 2017, the same low value (0.44) was noted in Central African Republic. Countries with high PHI values were generally stable over time. The maximum value of the index remained unchanged – approximately 0.9 in Japan. For several European countries (Iceland, Sweden, the Netherlands, Spain, Italy and France), there was a slight decrease in the index in 2017 compared to 2015.

The regions of Russia were homogeneous for a long time, with the index values within the country ranging from 0.6–0.8. In 1990 the worst value of the index was in Tuva (0.64), which is comparable to Uzbekistan, the Philippines, Vanuatu and Cape Verde. The value of the index in Moscow (0.75) was close to Tonga and Albania. The highest values (0.78) were in Dagestan and Karachay-Cherkessia that corresponded to United Arab Emirates, Kuwait and Czech Republic. After 2015, the index values changed to 0.8–0.9 in some regions: the largest cities of Russia (Moscow and St. Petersburg), the republics of the North Caucasus, several regions in the south of the European part of Russia (Belgorod, Voronezh, Krasnodar, etc.) and two Siberian oil and gas producing regions, the Khanty-Mansiysk and the Yamalo-Nenets autonomous regions. The maximum value of the index in 2017 in Ingushetia (0.90) was the same as in Cyprus, Denmark, or Germany.

### Observed trends in health indicators

**Fig. 2:**
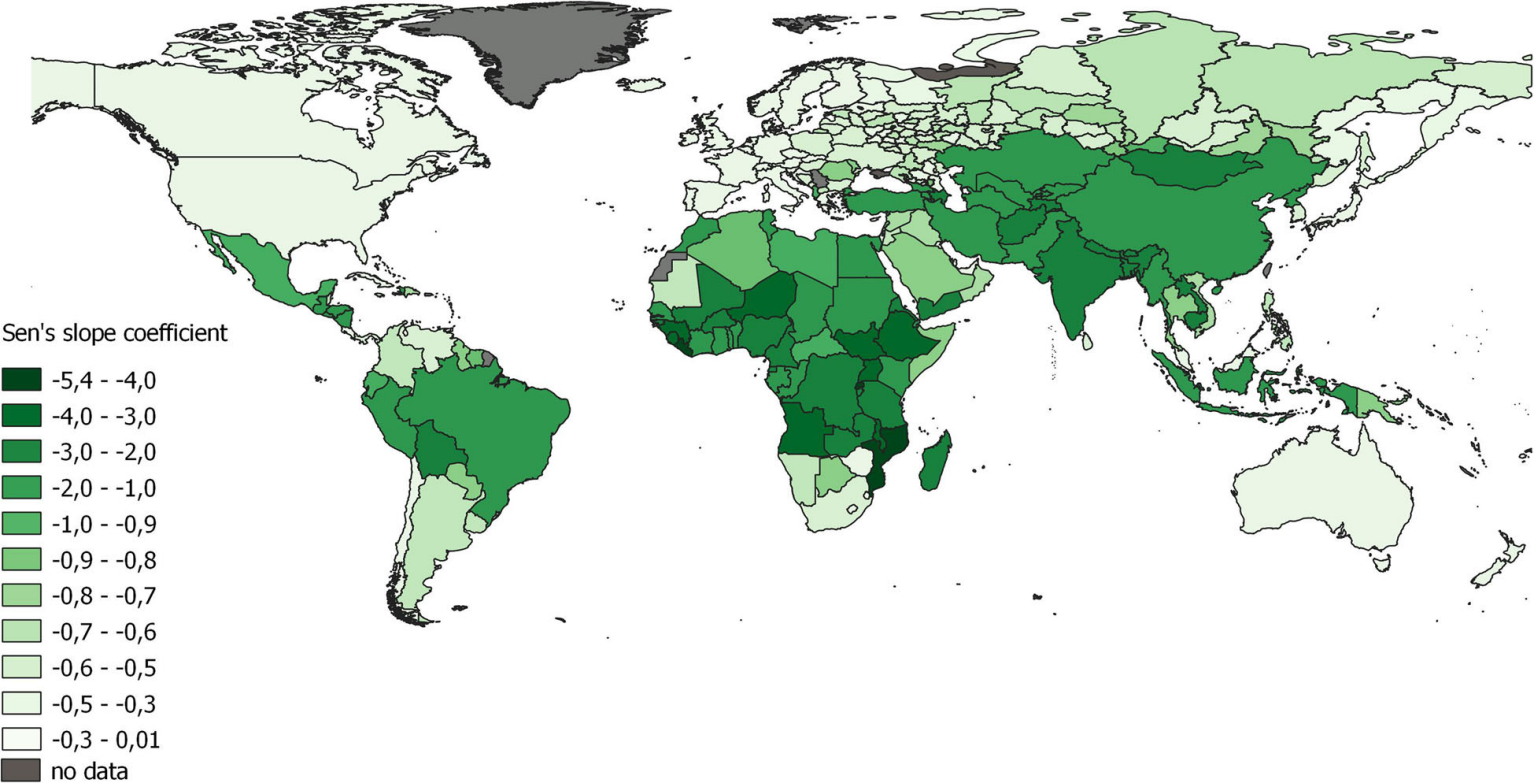
Decline in infant mortality according to the spatial distribution of Sen’s slope coefficients of trends in the regions Russia and in the world during 1990–2017

Since life expectancy and infant mortality determine the PHI, their spatiotemporal trends were analysed. The most obvious trends towards a decrease in infant mortality were found in African (the most intensive decline was observed in Sub-Saharan Africa with Sen’s slope coefficient below 4.0), Asian, and some South American countries (Fig. 2). The decline in infant mortality in regions of Russia was within a small range of Sen’s slope coefficient, from 0.3 to 0.9. The maximum rates of decline (0.98–0.70) were observed primarily in Siberian regions (Tyva, Altai, Khakassia and the Trans-Baikal Region), as well as in some republics of the North Caucasus (Ingushetia and Chechnya). At the same time, the coefficient values in Tuva corresponded to Ecuador, in Altai and Ingushetia – to Mexico and Lebanon, and in Khakassia and Trans-Baikal Region – to Romania, Guyana, Somalia. The minimum rates (0.35–0.40) were predominantly found in the European territories of Russia but were also observed in Kamchatka (Far East) and Karachay-Cherkessia (North Caucasus). It is comparable to Hungary, Lithuania, South Africa and Qatar. The changes in infant mortality in Russia were more noticeable than those in European countries but less noticeable than those in countries of Asia and South America. A sharp increase in infant mortality in Russia in 1993 and 2012 should be mentioned, it is associated with the transition to new criteria for live birth according to the WHO recommendations [54].

The trends were statistically significant (*p*-value < 0.05) for all countries except several in Sub-Saharan Africa (Zimbabwe, Lesotho, Swaziland, South Africa) and island countries (Fiji, Saint Vincent, and the Grenadines). In Russia, this trend was not significant for Chukotka (in the northern part of the Far East), which can be explained by the small population in the region.

**Fig. 3:**
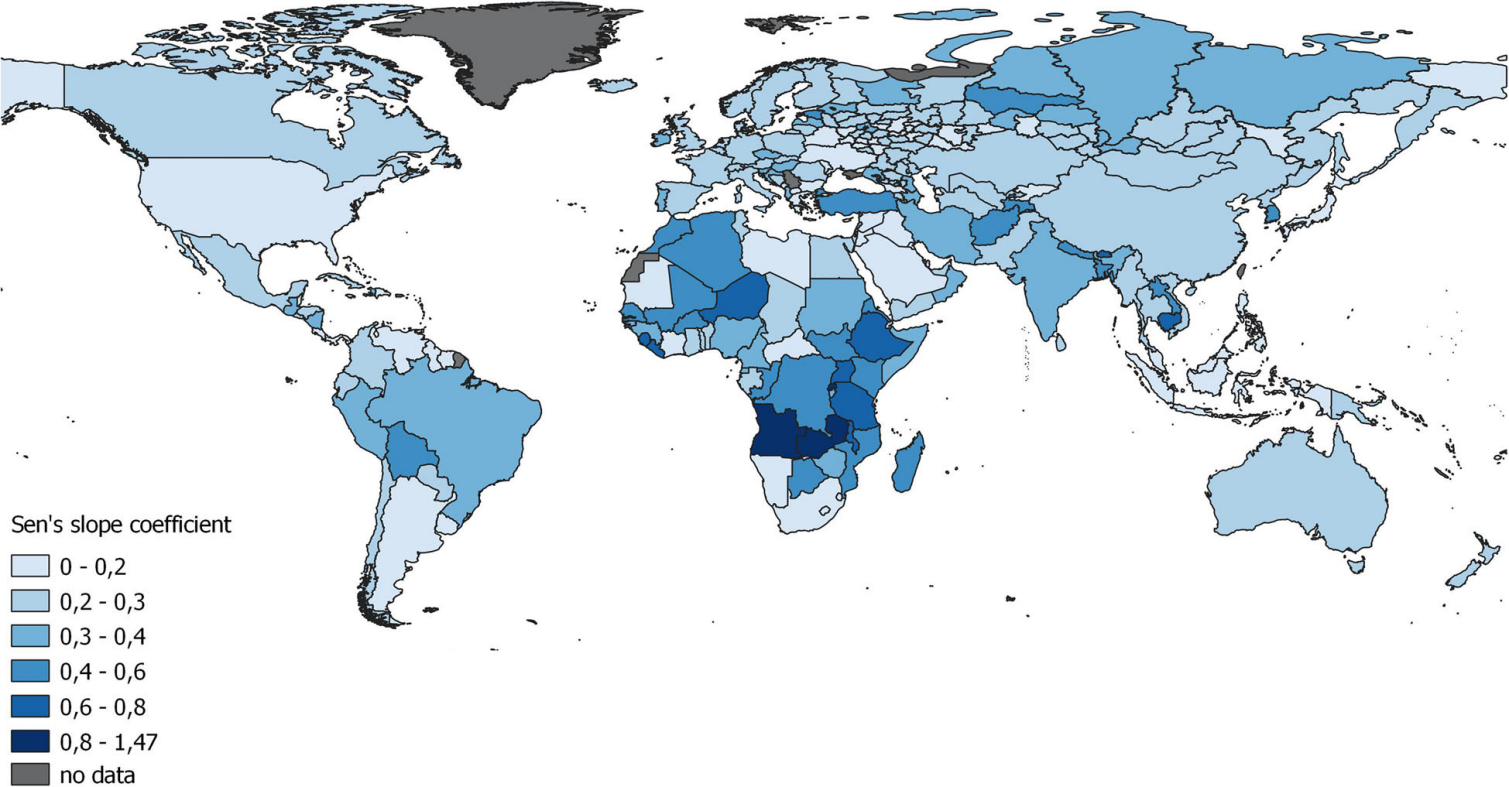
Increase in male life expectancy according to the spatial distribution of Sen’s slope coefficients of trends in the regions Russia and in the world during 1990–2017

As expected, trends towards an increase in male and female life expectancy were observed overall worldwide. The trend of increasing male life expectancy was most remarkable in Sub-Saharan Africa with Sen’s slope coefficient > 0.8 (Fig. 3). The variations in Sen’s slope coefficient for the regions of Russia were also small, ranging from 0.07 to 0.6. The most pronounced increase in life expectancy was recorded in three completely different regions – Ingushetia (North Caucasus), Moscow (the capital and largest city in the country) and the Khanty-Mansiysk autonomous region (an oil-producing region). The absence of changes was also identified in spatially dispersed regions – Mari El (Volga region), and the Amur region and Chukotka (Far East).

The trends were not statistically significant at the *p*-value < 0.05 in 13 analysed territorial units. These are some regions of the European part of Russia, and the Amur region and Chukotka in the Far East as well as Sub-Saharan African countries (Lesotho, Namibia, South Africa, and Swaziland), Syria and Belize. The number of such territories increased to 60 when statistical significance was set to a p-value< 0.001. They include 46 regions of Russia and Ukraine and Belarus.

**Fig. 4:**
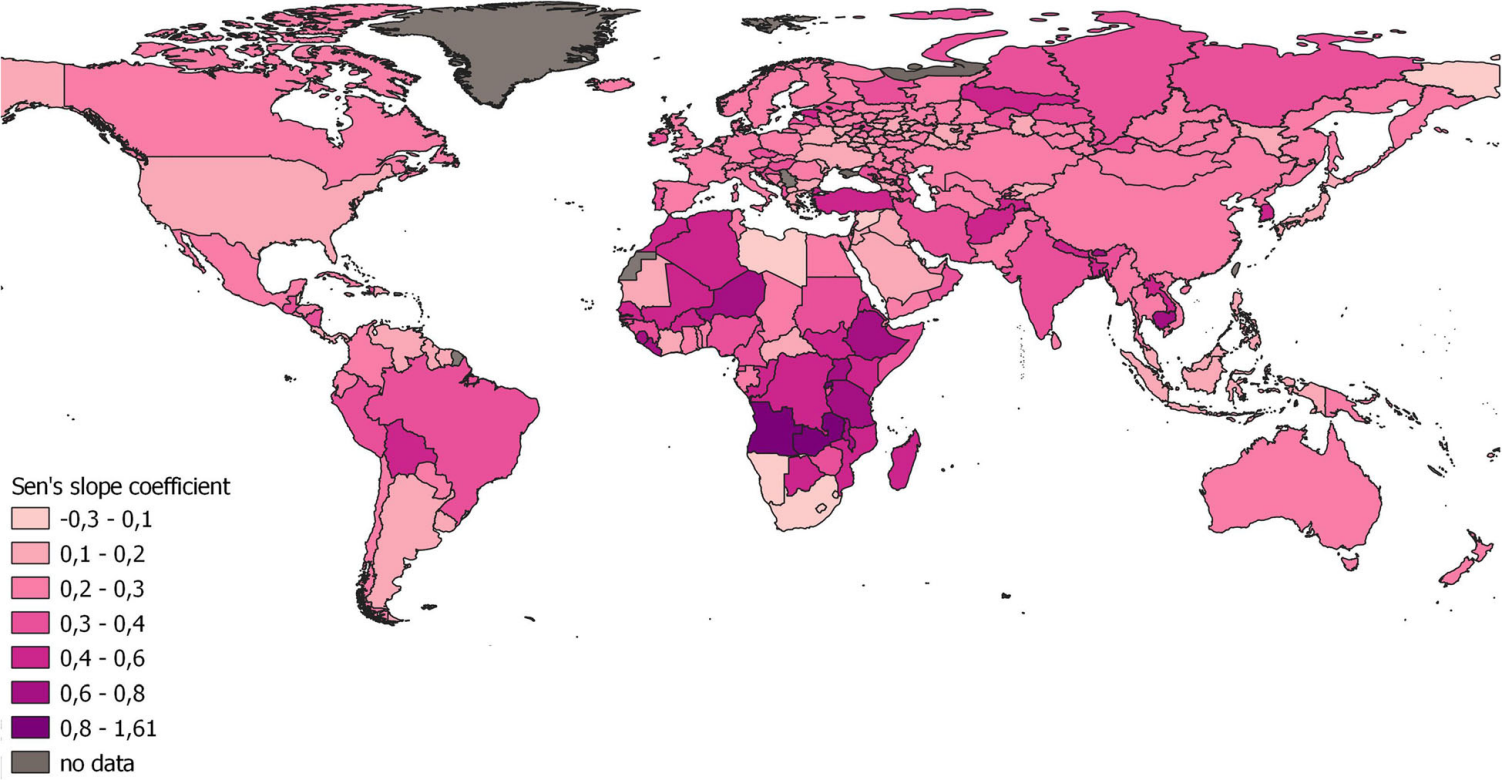
Changes in female life expectancy according to the spatial distribution of Sen’s slope coefficients of trends in the regions Russia and in the world during 1990–2017

The trends of female life expectancy were similar to the trends for male life expectancy. The Sen’s slope coefficient for the regions of Russia varies even less (from 0.06 to 0.4), although the range of values is from 0.18 to 1.61 at the global level (Fig. 4). The maximum increase in female life expectancy was identified in the same regions where the increase in male life expectancy was observed. There were no changes in female life expectancy in Chukotka, the Jewish Autonomous Region (Far East) or Chechnya (North Caucasus).

In addition, the trends of female life expectancy were more statistically significant than those of male life expectancy. There was no statistical significance at the *p*-value < 0.05 in 10 territorial units (Chukotka and Chechnya in Russia, as well as Sub-Saharan Africa, Belize in Central America). In total, 36 territorial units, including 22 spatially dispersed regions of Russia, Montenegro and Iraq had nonsignificant trends at a p-value < 0.001.

### Health and income level

**Fig. 5:**
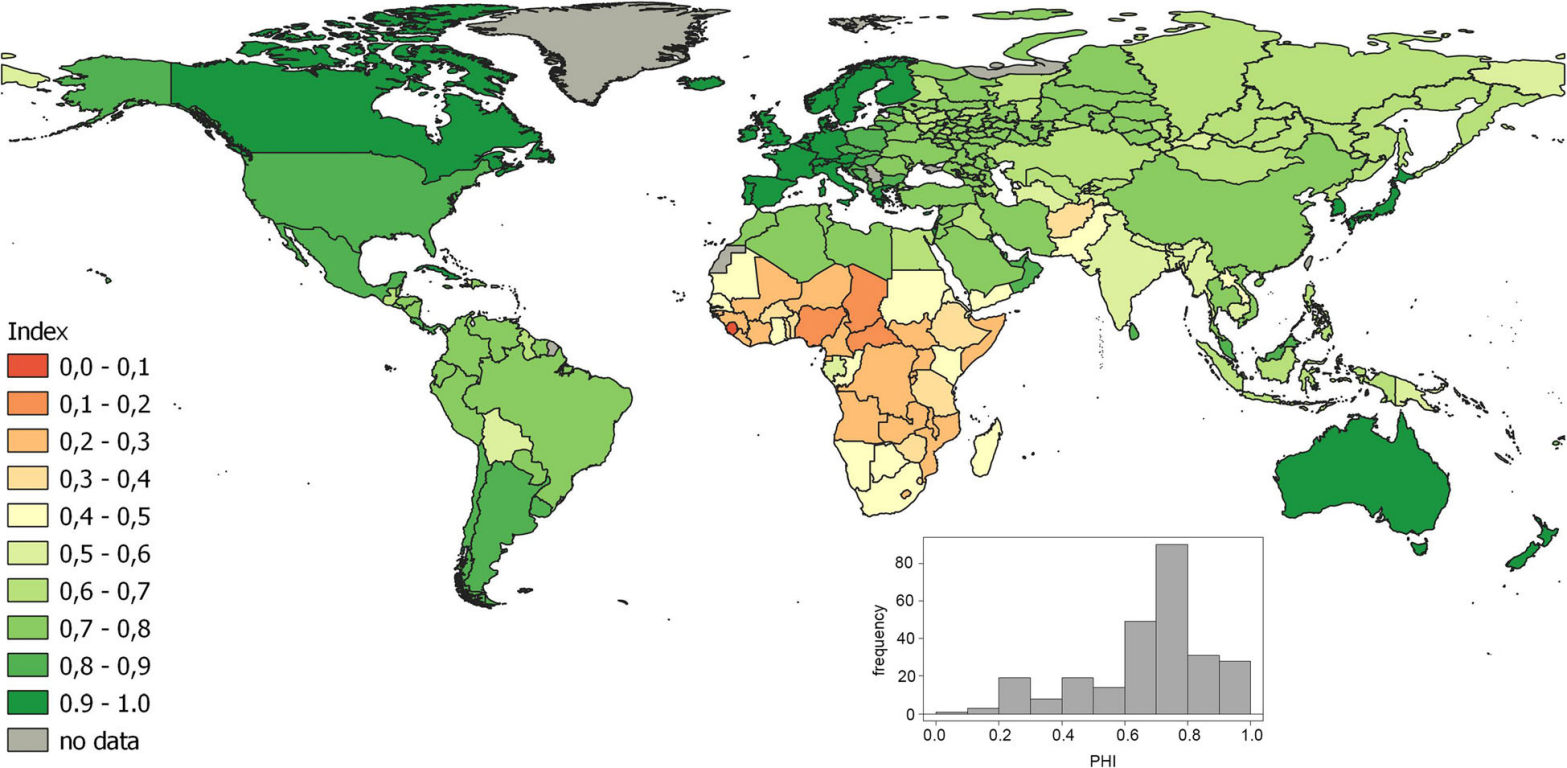
Spatial distribution of average PHI in the regions of Russia and in the world during 1990–2017

**Fig. 6:**
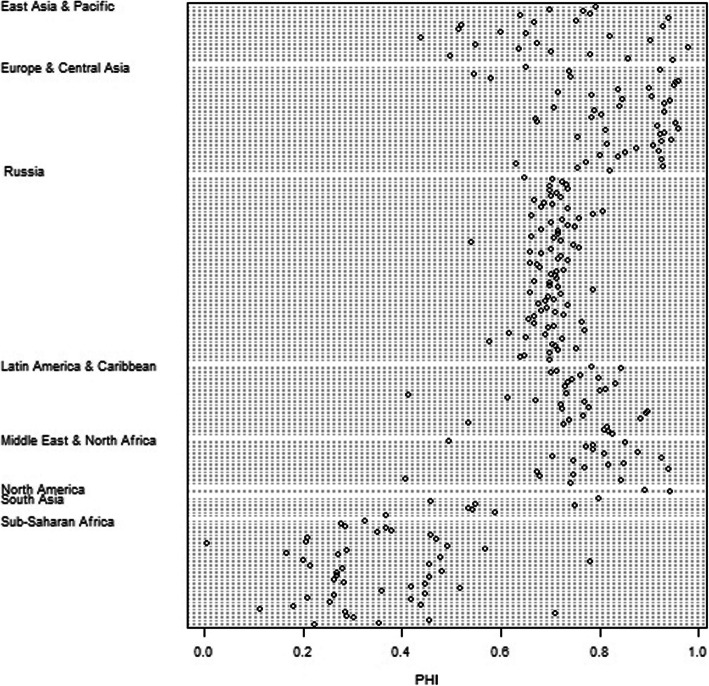
Distribution of PHI in regions of Russia and in countries according to the World Bank geographic regions

The PHI varied from 0.05 (Sierra Leone) to 0.98 (Japan), on average, during 1990–2017. Most territorial units were valued from 0.6 to 0.8 (Fig. 5). The PHI values were the most homogeneous in the Europe & Central Asia group^2^ and the least homogeneous in Sub-Saharan Africa. The regions of Russia were quite compact in the distribution of the PHI values (Fig. 6). The index values varied from 0.5 to 0.8. Moreover, the maximum PHI values in Ingushetia and Dagestan (North Caucasus) and the lowest PHI values in Tyva, the Jewish Autonomous Region and Chukotka (Siberia and the Far East) were defined as outliers.

After excluding outliers, the highest PHI values were recorded in Moscow and St. Petersburg and in the republics of the North Caucasus. The regions with high PHI are located in the south of the European part of Russia and in areas of oil and gas production in Western Siberia. The Far Eastern regions, Eastern Siberia, and the northern European part of Russia were identified as having the lowest PHI.

The regions of Russia were close to the regions of Latin America & the Caribbean and the Middle East & North Africa in terms of PHI values, although many of the regions of Russia had lower values. The PHI was predominantly higher in the European and Central Asian regions.

**Fig. 7:**
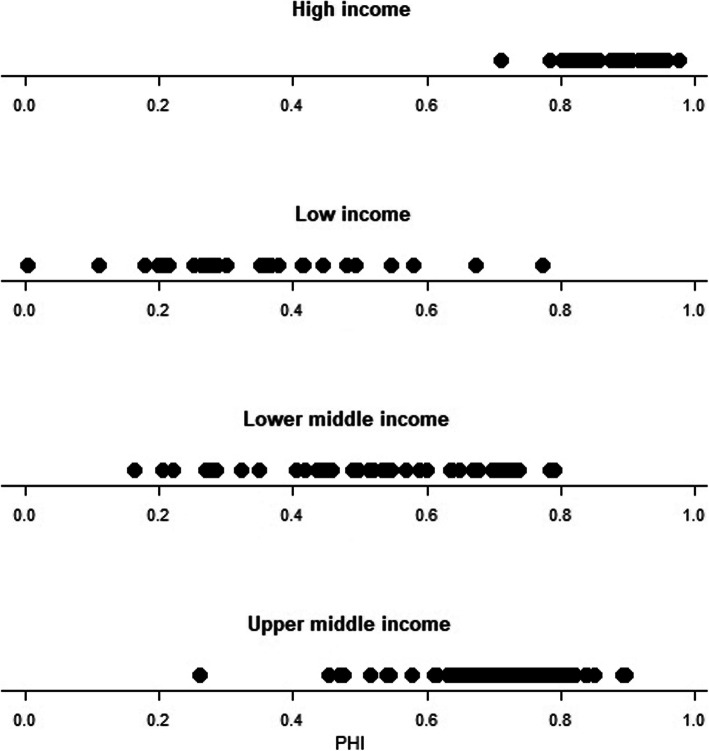
Distribution of PHI in countries according to the World Bank income groups of regions

The countries belonging to the high-income group^3^ were the most homogeneous in terms of the PHI (Fig. 7). A wide range of PHI values was observed in the low-income group and a slightly smaller range in the lower-middle-income group. The upper-middle-income group included the outliers.

The average PHI values corresponded with income level; however, there was a slightly lower PHI in regions of Russia (0.7) compared to other countries with upper middle income (0.73) (Table 1).

**Table 1:** Average values of the PHI by income level

Income group	Index (mean)	Index (standard deviation)	Numbers of territories
High income	0.89	0.06	51
Low income	0.34	0.16	31
Lower middle income	0.53	0.17	45
Upper middle income	0.73	0.12	54
Russia (Upper middle income)	0.70	0.04	81

**Fig. 8:**
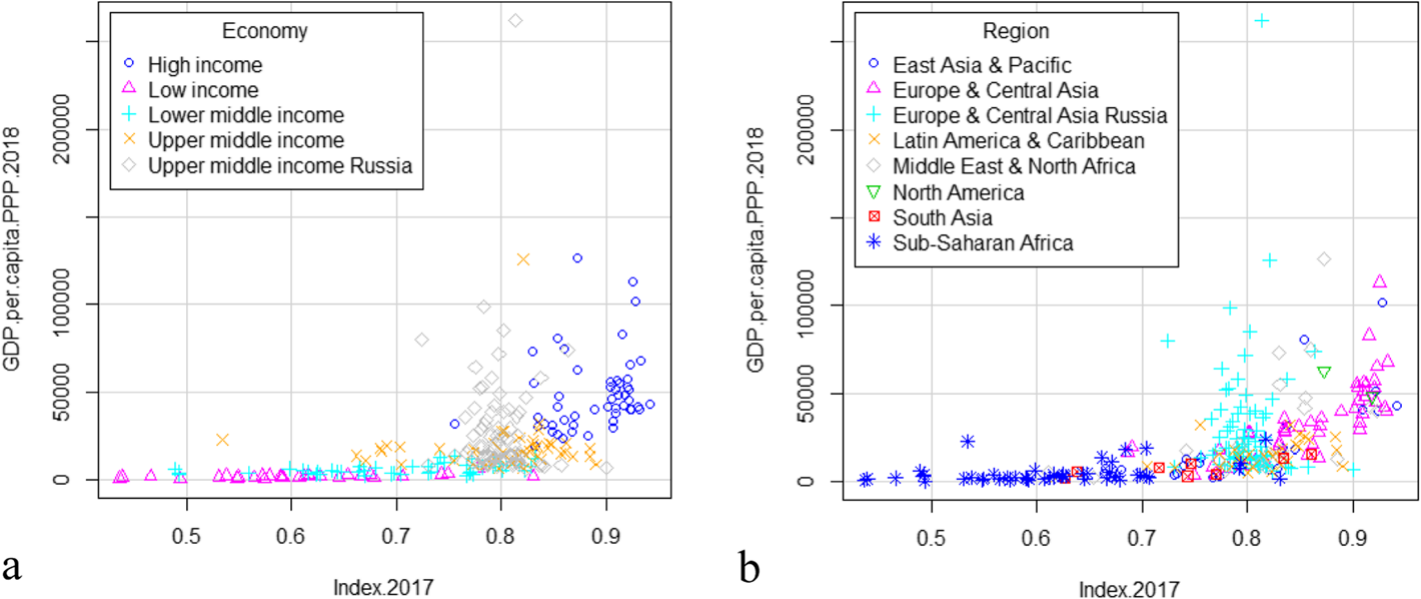
Distribution of PHI in regions of Russia and in countries in accordance with GDP (a) and World Bank geographic regions (b)

The distribution of the PHI depending on GDP per capita was analysed similarly. The GDP data for 2018 and the values of the PHI for 2017 were used. There was a general trend of an increase in the PHI with an increase in GDP. However, while many regions of Russia were comparable to the countries in the high-income group in terms of GDP per capita, they had a lower PHI (Fig. 8). Moreover, the regions of Russia had PHI values lower than those in many countries in Europe & Central Asia and the Middle East & North Africa, although they had comparable GDP per capita.

### Typological classification

**Fig. 9:**
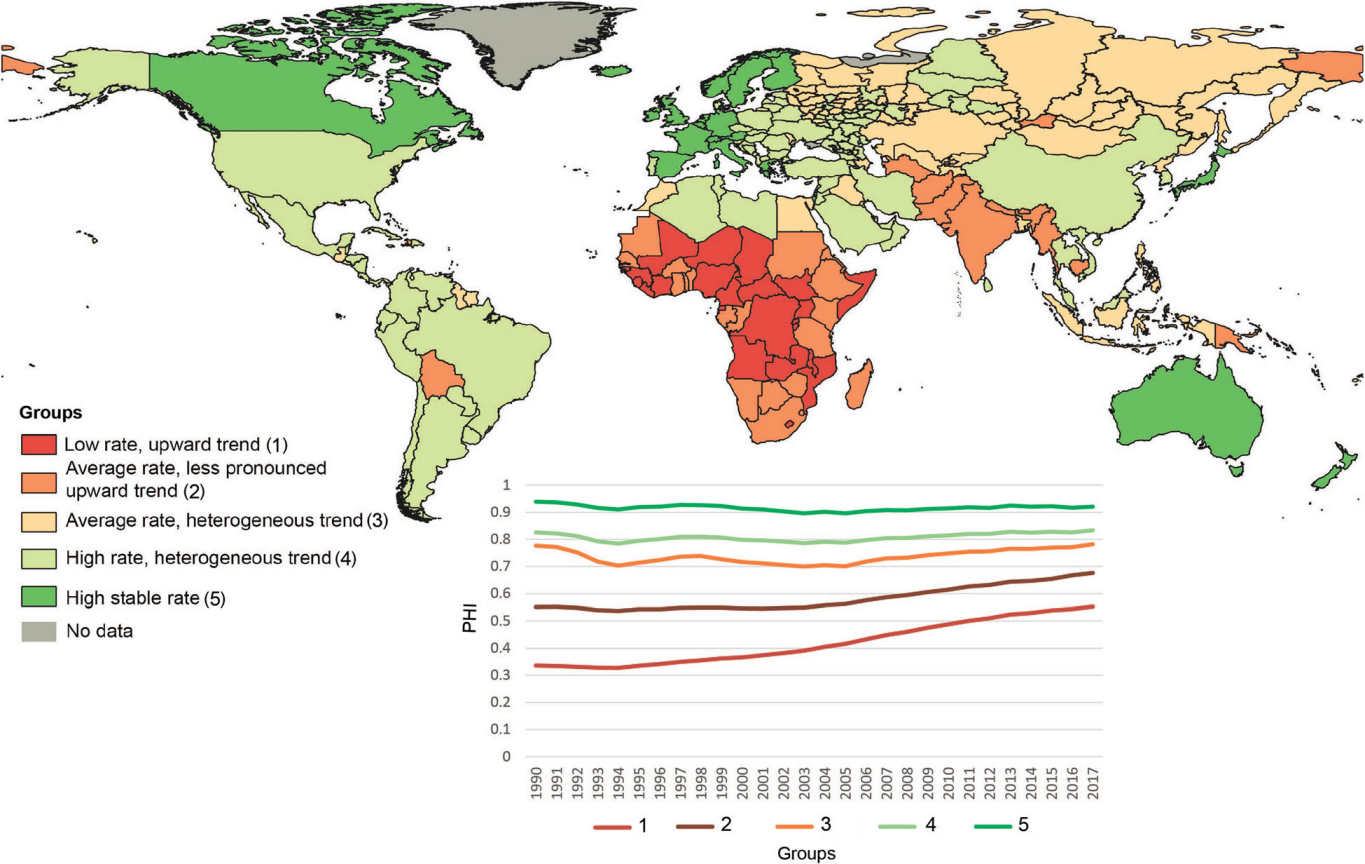
Typological grouping of regions of Russia and countries based on PHI

Five homogeneous groups of countries and regions of Russia were identified according to the PHI in 1990–2017 (Fig. 9).

*The first group* (low rate, upward trend) included 23 countries exclusively on the African continent. This group was characterized by the lowest values of the index (0.42) with a pronounced increase over time.

*The second group* (average rate, less pronounced upward trend) was characterized by slightly higher PHI values (0.58) than the previous group but with a less pronounced upward trend. This group included 36 countries located mainly in Africa and Asia and some Pacific island states. Additionally, this group included one state of the post-Soviet space – Turkmenistan – and two regions of Russia – Tyva and Chukotka.

*The third group* (average rate, heterogeneous trend) was distinguished by the most heterogeneous dynamics of the PHI over time. With an average value of 0.74, it fluctuated in selected years. There was a decline from 1990 to 1994, growth around 1998, another decline around 2005, and subsequent growth. This group included post-Soviet countries (Azerbaijan, Kazakhstan, Kyrgyzstan, Tajikistan, Uzbekistan, and Moldova), as well as 44 Russian regions (mainly in Siberia and the Far East).

*The fourth group* (high rate, heterogeneous trend) had an average index value of 0.81. There was also a noticeable tendency for a decrease by 1995 and subsequent stabilization over time. This was the largest group, with 108 territorial units, including countries of Central and South America, Southeast Asia (Malaysia, Vietnam, Thailand), the Arabian Peninsula (Bahrain, Brunei, Kuwait, Oman, Qatar, Saudi Arabia, the United Arab Emirates), and South Korea, China, Iran, and the United States. The same group included the former socialist European countries, some of the post-Soviet countries, Belarus, Ukraine, Armenia, Latvia, Lithuania, Estonia, and 36 regions of Russia (mainly the southern part of European Russia, as well as oil-producing regions in Western Siberia).

*The fifth group* (high stability rate) was characterized by the highest PHI values (0.92) and was stable over time. It included 24 countries, mainly Western European countries, Canada, Australia, New Zealand, Singapore and Japan.

## Discussion

The study showed a trend towards convergence in the health status in the world. Over the almost 30 years, the level of health of population has increased significantly, as evidenced by the reduction in the spread of the index values (the range of index values was from 0 to 1 in 1990 to 0.4–1 in 2017). This is largely due to the ongoing convergence of infant mortality since 1950 [55]. Our study shows that the trend towards a decrease in infant mortality is statistically significant almost everywhere, except in some African countries and island states. There was a relatively rapid decline in infant mortality in regions of Russia. In the opinion of Andreev (2020) values of infant mortality are still higher than in European countries with reliable statistics due to conditions arising in the perinatal period as well as congenital disorders [54]. This may be a consequence of both factors – a lower level of medical care for pregnant women and newborns in comparison with developed European countries, and unequal access to health care.

Life expectancy has a less stable trend towards convergence between countries due to the sensitive response of mortality, which determines life expectancy, to various crises and social shocks [56]. Worthy of note is the lack of statistical significance of the trend of increasing life expectancy in many regions of Russia, which is more pronounced for male life expectancy than for female life expectancy. The trend towards an increase in male life expectancy is nonsignificant in almost half of the regions of Russia by *p*-value< 0.001. This indicates periodic fluctuations in life expectancy over the past 30 years, which, in turn, may indicate the absence of a stable economic and social situation in the country.

**Fig. 10:**
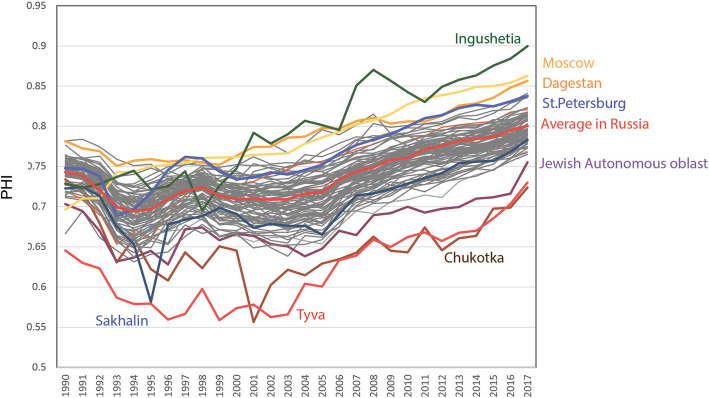
Changes in PHI in average in Russia and in regions in 1990–2017. Regions with the highest and lowest values are marked by colour

The greatest decline in the health level was noted in the early 1990s, when a sharp decrease in life expectancy due to an increase in mortality from heart disease and violent causes was observed in Russia and other post-Soviet countries (Fig. 10). The decline in the health level continued until 1994. Within the global experience of industrialized countries, this sharp decline in life expectancy represents a unique case [22], with rather strong regional differences [57]. In addition, the changes in health observed in those years cannot be explained solely by economic deprivation. The greatest causes were linked to the social environment, including an increase in reported crime and excessive alcohol consumption, as well as psychological stress and the deterioration of the health system [22, 57, 58].

After 1994 and until 1998, the health level began to rise again. However, in 1999–2005, there was a slight decrease and stagnation of the index, apparently associated with the recovery from the economic crisis of 1998. Only in 2007 did the health level in Russia return to the level of 1990 and begin to exceed it due to constant growth. It should be noted that the economic crises of 2008–2009 and 2014–2015 [59] are not visually reflected in the dynamics of the PHI.

Until the mid-1990s, the Russian regions were characterized by closer index values than those observed in the present. Strong fluctuations were typical for peripheral regions, such as Tyva, Chukotka and the Jewish Autonomous Region, and for Ingushetia, the North Caucasus region with the leading PHI.

The significant interregional heterogeneity in Russia is clearly evident in the types of PHI dynamics, and the regions can be classified into three of the five groups observed worldwide. The European, Siberian, and Far Eastern regions display a clear division. Thus, most of the regions in southern Russia are similar to the European countries of the post-Soviet space, where the PHI stabilized after 1995, while the Siberian and Far Eastern regions present a tendency similar to that of the post-Soviet countries of Central Asia, with large fluctuations in the index values. Tyva and Chukotka are comparable to poor African countries in terms of PHI.

Thus, strong spatial inequality in health of population was found in Russia. Similar patterns were found for the city of Natal in Brazil, where the difference in life expectancy between districts reached 25 years, and the districts with the worst indicators were comparable to countries in Africa [60]. In Russia, such inequality can be explained by the country’s economic geography, combined with a population decline, an ageing workforce, and a constant need to adapt to a series of economic crises [61]. Changes in the sectoral structure, including a boom in the oil industry, led to the rapid development of certain regions, while the development of many other regions slowed down.

**Fig. 11:**
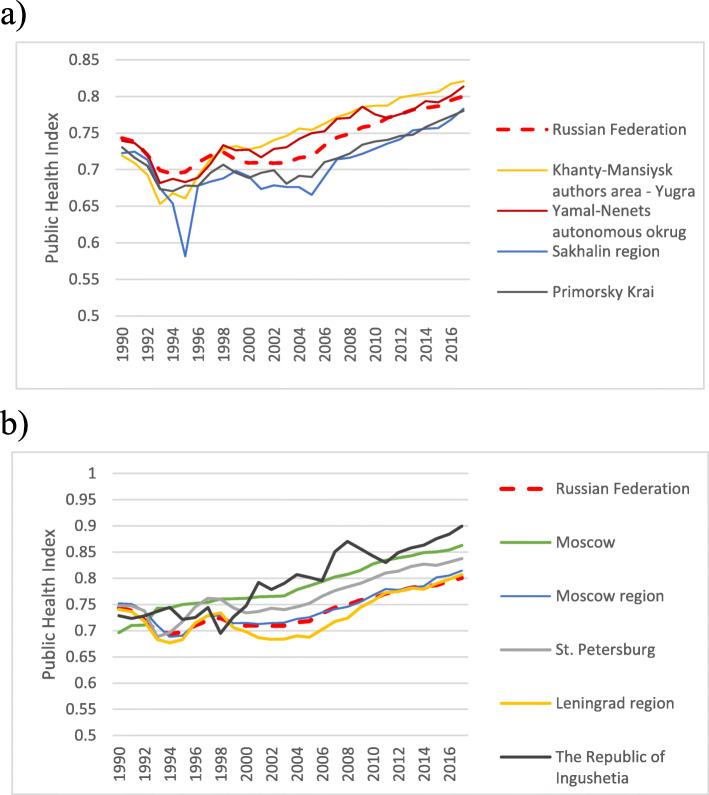
Changes in PHI in various regions of Russia – oil producing and Far East regions (a), main cities Moscow, St. Petersburg, and Ingushetia, region with the highest PHI values during 1990–2017 (b)

A specific change in the health level was observed in Khanty-Mansiysk, one of the oil-producing regions with severe climatic conditions. The PHI was below the national average and the average of many other regions in the 1990s (Fig. 11). After 1998, the situation changed dramatically, and the health level rapidly increased. During the same period, Sakhalin and Primorsky Krai in the Far East had health levels comparable to that of Khanty-Mansiysk in the 1990s, but until the present, their PHI values are still below the national average. Thus, the economic focus on the oil and gas sector and investment inflow could lead to a rapid improvement in the health of population in oil-producing regions, including those with severe climatic conditions.

There is also a difference between the two largest cities in the country, Moscow and St. Petersburg, and a significant gap vis-à-vis the surrounding regions, the Moscow and Leningrad regions (Fig. 11). Again, this difference was more noticeable in the 2000s than in the 1990s. Against this background, the positive dynamics of the PHI in Ingushetia in the North Caucasus look rather foreign.

Population health and GDP are closely interrelated indicators. The issue is that it is not just an increase in GDP can result in a health improvement [62, 63], but progress in national health can also lead to economic growth [64–66]. We found the discrepancy between the PHI and GDP per capita in Russia that are in line with other studies [45, 67]. Shkolnikov et al. (2019), identified the gaps between the observed and the Preston-expected life expectancy values (so called Preston-curve) for both Russia and Moscow [67]. Compared to countries with a similar income level, the main cause of this gap was due to mortality for external causes in the working-age population and cardiovascular disease at older ages [68]. Thus, mortality trends that have been identified in Russia for a long time, continue to be relevant even despite the progress in healthcare. The health status in Russia may be significantly higher according to GDP values. At the same time, rates of progress can be only partially reflected by GDP, without considering inequalities in income, education, and other social determinants [69, 70]. An unequal access to health care can be significant. Disparities should be eliminated not only between regions, but also within those regions. The discrepancy between economic indicators and health status is commonly observed in the regions of Russia [67]. Thus, Sakhalin is comparable to Singapore and Ingushetia to Honduras in terms of GRP per capita [61], and vice versa in terms of the PHI. The reason for this discrepancy may be linked to intraregional inequality. Spatial inequalities in health have also been confirmed by previous studies [67, 68, 71, 72]. There is no doubt that for countries as geographically expansive as Russia, inequality is determined not only by economic factors or healthcare costs, but also by climatic, ethnic and sociocultural diversity [73]. Determining the contribution of possible factors to the health status in Russia is a goal for future research.

Thus, a way out of the crisis of the 1990s in Russia can be observed only at the end of the 2000s. Bridging the gap with developed countries is still a challenge for the future despite the improvement in health status. In 2015, only six regions, with the exceptions of Moscow and St. Petersburg, approached comparable values of the index. In 2017, their number increased significantly with the addition of the regions of southern European Russia, the Volga region, and western Siberia. The consequences of the 2020 crisis have yet to be assessed, but it can be assumed that the trend towards an increase in the level of health of population in Russia will again slow down.

### Limitation

The main limitation of the study is the possibility of using regional data for international comparison. Such data, especially regarding socioeconomic characteristics, are very limited in Russia. Another limitation is the lack of high-quality municipal statistical data for intraregional research on a national scale. Main problems of data quality in Russian statistics are described in the introduction.

## Conclusion

The prolonged crisis of the 1990s caused the health status in Russia to be unsatisfactory for almost 20 years. Until the present, there has been a long-term tendency of a more favourable situation in the southeastern European part of the country and in the largest cities, Moscow and St. Petersburg, and a disadvantaged situation in the northern European part and a worsening of the situation in the east. In general, the regions in the European part are quite different from the Asian part of the country in terms of the level and dynamics of health. They are more similar to the European countries of the post-Soviet space, while the Siberian and Far Eastern regions present a tendency similar to that of the post-Soviet countries of Central Asia. In most of regions in Russia, the rate of change in population health has remained significantly slower. We found the discrepancy between population health and GDP per capita in Russia. While many regions of Russia were comparable to the countries in the high-income group in terms of GDP, the progress in health was less pronounced. Perhaps this can be explained by intraregional inequality, expressed by significant fluctuations in income levels, as well as unequal access to health care within the region. Further progress is impossible without focusing on the problem of intraregional inequality.

## Data Availability

All documents and publications in Russian are available at the Lomonosov Moscow State University, Moscow, Russian Federation.

## Abbreviations

PHI: Public Health Index

## Footnotes

1 Crimea and Sevastopol were excluded from the study due to the lack of statistical data for most of the period.

2 classification of countries by world regions according to the World Bank

3 World Bank classification
